# Survivin and vascular endothelial growth factor are associated with spontaneous pulmonary metastasis of osteosarcoma: Development of an orthotopic mouse model

**DOI:** 10.3892/ol.2014.2556

**Published:** 2014-09-24

**Authors:** LI ZHANG, YOUYOU YE, DEJIAN YANG, JIANHUA LIN

**Affiliations:** 1 College of Orthopedics and Traumatology, Fujian University of Traditional Chinese Medicine, Fuzhou, Fujian 350122, P.R. China; 2 First Affiliated Hospital of Fujian Medical University, Fuzhou, Fujian 350005, P.R. China

**Keywords:** orthotopic mouse model of osteosarcoma, survivin, spontaneous pulmonary metastasis, vascular endothelial growth factor

## Abstract

The high rate of pulmonary metastases of osteosarcoma (OS) presents a therapeutic challenge in the field of orthopedics. Therefore, there is a marked requirement to establish a spontaneous pulmonary metastasis animal model of OS, within which potential antitumor agents may be evaluated for their ability to inhibit the growth and pulmonary metastasis of OS, as well as to identify potentially associated biomarkers of OS metastasis. In the present study, rodent OS cells (UMR106-01) were injected into the right tibia of athymic nude mice. The mice were sacrificed weekly by cervical dislocation at one to five weeks following inoculation. The orthotopic mice developed tumor masses in the right tibia one week following inoculation. At three weeks, multiple nodules were observed in the lungs. The expression of survivin and vascular endothelial growth factor (VEGF) was analyzed in the tumors and lungs via immunohistochemistry. The positive expression of survivin and VEGF was identified in the local tumor and lung tissue of the orthotopic mice, however was not observed in the tissues of the healthy control mice. The orthotopic model established in the current study presents a valuable tool for the investigation of factors that promote or inhibit OS growth and/or metastasis. In addition, it was identified that survivin and VEGF may be significant in the lung metastasis of OS.

## Introduction

Osteosarcoma (OS) is the most common type of primary bone malignancy, which occurs during adolescence, as well as sporadically in adulthood (1,2). Although modern combination therapies have markedly improved, in the past 30 years, the five-year survival rate for patients with OS remains at ~60–70% (3,4). Mortality as a result of OS is usually due to pulmonary metastases, as in the majority of cases the local disease may be surgically treated. At presentation of OS, ~20% of patients already exhibit pulmonary metastases and almost all patients with recurrent OS demonstrate metastatic disease (5,6). Patients exhibiting metastasis or disease recurrence are associated with a long-term survival rate of <20% (7,8). Over the past 20 years, the understanding of OS metastasis has improved significantly, however, this has not been translated into substantial therapeutic advances or clinical outcomes. Therefore, there is a marked requirement for the development of a clinically relevant animal model to further investigate the mechanism(s) involved in OS metastasis.

Survivin is a unique member of the inhibitor of apoptosis protein family, which interferes with post-mitochondrial events, including the activation of caspases. Furthermore, survivin regulates the cell cycle (9), and functions to inhibit apoptosis, promote cell proliferation and enhance angiogenesis (10,11). Notably, while it is expressed in almost all malignancies, survivin is rarely detected in healthy differentiated adult tissues (12). In OS, survivin is overexpressed and is considered to be important in protecting cells from apoptosis (13), however, the correlation between survivin expression levels and lung metastasis of OS remains unknown.

Vascular endothelial growth factor (VEGF) is a well-known angiogenic factor, which is important for vascular development and maintenance in all mammalian organs (14), and is also involved in tumor angiogenesis and metastasis (15). VEGF expression has been found to correlate with the prognosis and metastasis of OS (16,17). Furthermore, VEGF expression is often detected in tumoral tissues; however, its involvement in spontaneous metastatic lung cancer is poorly understood.

In the present study, an orthotopic mouse model was developed to investigate the mechanisms associated with OS metastasis. Using this model, the protein expression levels of survivin and VEGF of *in situ* tumors and metastatic lungs of mice were analyzed. The aim of the present study was to establish a novel model within which to test the efficacy of treatment options for metastatic OS and to identify a potential tool to determine factors which promote or inhibit the growth and metastasis.

## Materials and methods

### Cell culture and animal maintenance

The UMR106-01 rat OS cell line was cultured in high-glucose Dulbecco’s modified Eagle’s medium supplemented with 10% fetal bovine serum (HyClone; Thermo Fisher Scientific, Rockford, IL, USA) and antibiotics at 37°C in an atmosphere of 5% CO_2_. A total of 30 four-week-old male Balb/c nu/nu mice (Shanghai Laboratory Animal Co., Ltd., Shanghai, China) were purchased and housed in the animal center at Fujian University of Traditional Chinese Medicine (Fuzhou, China). The mice were acclimatized for one week prior to experimental manipulation. The care and use of the mice followed the approved guidelines of the Institutional Animal Care and Use Committee and was approved by the ethics committee of Fujian University of Traditional Chinese Medicine (Fuzhou, China).

### Orthotopic intratibial injection

OS cells were injected into the right tibiae of mice, as described previously (3,18). Specifically, 25 mice were anesthetized with 4% chloral hydrate (0.1 ml/10 g), and the right leg was cleaned using 75% ethanol. Cultured OS cells were harvested, counted and re-suspended in phosphate-buffered saline (PBS) at a final concentration of 5×10^6^ cells/ml. The cortical layer of the bone was pierced using a 25-gauge needle (Zhejiang KangKang Medical-Devices Co., Ltd., Zhejiang, China), which was then inserted ~3–5 mm into the diaphyseal shaft of the tibiae for the delivery of 5×10^5^ OS cells in 100 μl PBS. The left limb of each mouse was inoculated with PBS and served as a control.

### Primary tumor growth and pulmonary metastases

Tibial tumor growth was measured weekly using a micrometer for five weeks, beginning one week following inoculation. The tumors grew to be almost spherical ellipsoids. The tumor diameter (skin to skin) was measured in two perpendicular dimensions (D1 and D2), and the tumor volume was calculated according to the following formula: Tumor volume = 4/3π [1/4 (D1 + D2)]^2^. Five mice were sacrificed by cervical dislocation at one, two, three, four and five weeks following inoculation. Tumor size and pulmonary metastases were recorded weekly. The primary tumors in the tibia were excised and stored in 4% paraformaldehyde. In addition, the lungs were excised and stored in 4% paraformaldehyde following inspection with a microscope (BX51, Olympus Corporation, Tokyo, Japan).

### Histology

The fixed tumor masses from the tibiae were embedded in paraffin and sectioned at 5 μm. The sections were stained with Harris’ hematoxylin and eosin (H&E) for histological analysis, according to general laboratory instructions. The lung specimens were also embedded and sectioned to 5 μm, whereby every fifth section was stained with hematoxylin and eosin for tumor screening.

### Immunohistochemistry (IHC)

A standard IHC method was adopted to stain the tumor and lung sections. Polyclonal rabbit anti-rat survivin (Santa Cruz Biotechnology, Inc., Santa Cruz, CA, USA) and polyclonal mouse anti-rat VEGF antibodies (Santa Cruz Biotechnology, Inc.) were used. The samples were then incubated with horseradish peroxidase-conjugated secondary goat anti-rabbit and goat anti-mouse polyclonal antibodies (Envision; Dakocytomation, Shanghai, China) and liquid 3,3’-diaminobenzidine+ (Dako NorthAmerica, Inc., Carpinteria, CA, USA) was used for detection. The sections were subsequently counterstained with H&E. The right tibiae of the control group were fixed and decalcified in 10% EDTA for two weeks, and the solution was replaced every second day (3). The tibiae from mice in the treatment group did not require decalcification, as the tibia was almost completely replaced by the tumor. Decalcified right tibiae and lungs of a healthy mouse were also stained for survivin and VEGF using the aforementioned antibodies and served as a negative control, with the PBS-inoculated left limbs of each mouse serving as non-specific controls.

## Results

### Characterization of the orthotopic mouse model of OS

**Figure 1:**
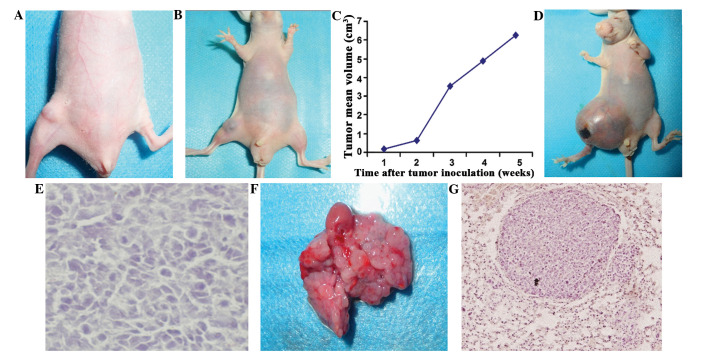
The macroscopic appearance of the orthotopic primary tumor from mice (A) one and (B) two weeks following inoculation. (C) A tumor growth curve, which presents the mean volume of the orthotopic primary tumors at various time points. (D) Macroscopic appearance of the orthotopic primary tumor five weeks following inoculation. (E) Histological characterization of the orthotopic primary tumor (stain, hematoxylin and eosin; magnification, ×200). (F) Multiple nodules were observed in the lungs at five weeks. (G) Histological characterization of one of the nodules visible in the lungs at five weeks (stain, hematoxylin and eosin; magnification, ×200).

Tibial tumors were observed macroscopically at the first week following inoculation (Fig. 1A; Table I). The tumor size had increased rapidly two weeks following inoculation (mean tumor volume, 0.76 cm^3^) and the skin overlying the tumor was red (Fig. 1B and C). After five weeks, a large, local tumor mass (mean tumor volume, 6.3 cm^3^) was observed (Fig. 1D). Microscopically, the tumor exhibited numerous pleomorphic matrix components consisting of osteoblasts and pleomorphic tumor cells (Fig. 1E). No lung metastases were identified macroscopically after one or two weeks. However, macroscopic nodules were visible in the lungs at weeks three (4/5 mice), four (5/5 mice) and five (5/5 mice) (Fig. 1F and G; Table I).

### Survivin and VEGF expression in tumors and metastatic lungs

**Figure 2:**
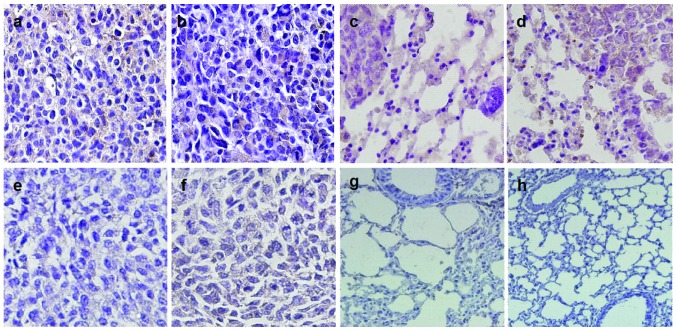
(A) Survivin and (B) vascular endothelial growth factor (VEGF) expression in the five-week primary tumor. (C) Survivin and (D) VEGF expression in the five-week lungs. No (E) survivin or (F) VEGF expression was identified in the healthy bone. No (G) survivin or (H) VEGF expression was identified in the healthy lungs (stain, hematoxylin and eosin; magnification, ×200).

IHC revealed positive survivin and VEGF expression in the tibial tumors (Fig. 2A and B), and pulmonary metastases (Fig. 2C and D), which was predominantly observed in the cytoplasm of the tumor cells. The healthy mouse control samples exhibited no survivin and VEGF expression in the tibiae (Fig. 2E and F) or the lungs (Fig. 2G and H). The experiments were repeated three times and similar results were obtained.

## Discussion

The high rate of pulmonary metastases of OS and the intolerance of patients to the toxicity levels experienced during current treatment regimes presents a therapeutic challenge in the field of orthopedics. Consequently, molecular targeted therapies are becoming a focus of cancer research with the aim of identifying future therapeutic treatment strategies (19). Therefore, the identification of proteins that are associated with the progression and metastatic nature of OS is critical. In the present study, a spontaneous pulmonary metastases orthotopic model of OS was developed using UMR106-01 cells. In contrast to previous studies (3), a high rate of *in situ* tumor formation and pulmonary metastases was observed following inoculation (Table I). The difference between the results of the present study and those of previous studies may be attributed to the method, as the cells were re-suspended in PBS and an increased concentration of cells was injected (5×10^6^ cells/ml) during the present study. The orthotopic model used in the current study presents a valuable tool for investigating the biomolecules that promote or inhibit OS growth and/or metastasis, and may also be used to assess the effect of anti-target agents, which are specific to OS.

Survivin inhibits apoptosis, and promotes proliferation and angiogenesis, which are key components of tumor growth and metastasis. While previous studies have shown that survivin is involved in the inhibition of apoptosis in OS (20,21), few studies have investigated the correlation between survivin and pulmonary metastasis. In the present study, survivin was observed to be expressed in the metastatic lungs, indicating that survivin may be associated with spontaneous pulmonary metastases. Previous studies have identified a positive correlation between angiogenic stimulation and vascular endothelial cell growth, and survivin expression. Furthermore, survivin, in addition to AKT, is known to activate anti-apoptotic signaling pathways that lead to vascular endothelial cell growth and tumor angiogenesis (22). These results facilitate the elucidation of the possible role of survivin in pulmonary metastases of OS. An elevation in the levels of VEGF expression was also identified in the metastatic lungs. VEGF is involved in pulmonary metastases of OS and its elevated expression may also be associated with spontaneous pulmonary metastases of OS. As survivin promotes the growth of vascular endothelial cells, it is hypothesized in the present study that survivin may affect the secretion of VEGF, however, further study is required to investigate this theory.

In conclusion, the present study successfully established a novel spontaneous pulmonary metastases orthotopic mouse model of OS. Using this model, the expression levels of survivin and VEGF were identified in the metastatic lungs, indicating that these two factors may be important in OS metastases. These results have a potential clinical implication in the prediction of metastasis and in the development of novel combined targeted therapies for the treatment of OS.

## Figures and Tables

**Table I:** Transplantation and spontaneous metastasis of UMR106-01 cells orthotopically injected into nude mice.

Time after tumor inoculation, weeks	Tumor-take rate at inoculation site	Mice with microscopically visible lung metastases, n	Mice with macroscopically visible lung metastases, n
1	5/5	0/5	0/5
2	5/5	4/5	0/5
3	5/5	5/5	4/5
4	5/5	5/5	5/5
5	5/5	5/5	5/5

## Abbreviations

OS: osteosarcoma
VEGF: vascular endothelial growth factor
